# Exploring a competency framework for the chief financial officer of a hospital: a qualitative study from China

**DOI:** 10.1186/s12913-023-09711-1

**Published:** 2023-06-27

**Authors:** Hongzhi Wang, Xin Xiang, Luping Dong

**Affiliations:** 1 Research Center of Hospital Management and Medical Prevention, Guangxi Academy of Medical Sciences, The People’s Hospital of Guangxi Zhuang Autonomous Region, Nanning, Guangxi 530021 China; 2 Institute of Fiscal and Finance, Shandong Academy of Social Sciences, Jinan, China; 3 Department of Neurology, The People’s Hospital of Guangxi Zhuang Autonomous Region, Nanning, China

**Keywords:** CFO, China’s hospital, Competency framework, Management, Leadership, Qualitative research

## Abstract

**Background:**

Hospital chief financial officer (CFO) plays a vital role in supporting the effective management of organization. Understanding their competencies is essential to improve hospital development and health care services in China. This paper aims to explore competencies necessary for hospital CFOs to fulfil their management responsibilities and develop a competency framework for hospital CFOs in China.

**Methods:**

A qualitative study was applied by conducting in-depth interviews with 151 participants from 15 Chinese provinces, comprising 89 individuals from 67 hospitals, and 62 individuals from 39 medical universities. Interviews were anonymised, recorded and transcribed. Qualitative thematic analysis was applied through a multi-stage review process and modified via the Delphi process using a national panel of 36 experts.

**Results:**

Using content analysis, we identified 17 competencies organized into three themes (personal attitudes, leadership competencies and managerial competencies) to conduct a competency framework for hospital CFO to fulfil their management practices. Those competencies emphasized the integration of different competencies required by the hospital CFO.

**Conclusions:**

This paper identified the detailed expertise, abilities and personal traits required by hospital CFOs in China, expanding the insights and perspectives of hospital CFOs currently working in China to literature. The proposed framework will help hospitals establish selection criteria, coaching tools, and development plans for CFOs.

## Background

Chief financial officers (CFOs) play a pivotal role in dealing with organization environments, strategy management and resources allocation [1–3]. Most studies have focused on corporate CFOs [4–7], but there has been less research on hospital CFOs. In the Chinese context, with the growing complexity of the health system environment and the demands of health services, hospitals began to develop the CFO position, expecting them to demonstrate capabilities to meet the demands of the position. However, our knowledge of CFO competencies in this context is limited. This paper aims to address this unfilled space and proposes an exploratory study to identify competencies required by CFO to fulfil their responsibilities in China’s hospitals.

The CFO role is established through appointment, serving as an oversight function for an organization’s financial matters and reporting. Typically, the CFO holds a leadership position as the second-in-command within the organizational hierarchy [8]. In corporate settings, the CFO’s responsibilities often include analysing the performance of business units, engaging in strategic planning, and handling stock exchange reporting [9]. Scholars have identified four roles of the CFO, including finance expert, generalist, performance leader, and growth champion [10]. Non-profit organizations, such as health care, education and social services, are increasingly assuming responsibility for services that were traditionally considered the domain of the government [11]. Non-profit CFOs may face challenges due to the comparatively lesser emphasis historically placed on the economic approach underlying accounting for non-profit organizations [12]. Therefore, the responsibilities of CFOs and the competencies required by them to perform their roles vary with the type of organization.

In addition to efficiently and autonomously handling cost control and performance budgeting matters [13], CFOs from health service organizations play a significant role in shaping the strategic direction of their respective organizations [14]. Advanced medical systems have previously established the position of CFO to improve management of the health care system by playing pivotal roles in corporate leadership and management, stewardship and accountability, financial management, commercial acumen, professional leadership and management. For example, NHS CFOs play a crucial role in the efficient delivery of safe, effective and financially sustainable services by providing strategic focus, corporate management, decision-making and leadership; additionally, they bear the responsibility of establishing the overall financial direction of the institution and fostering a culture that promotes collaboration, innovation, quality, and cost-effectiveness while ensuring prudent and efficient use of public funds [15], thus needing practical capabilities and technical knowledge, such as communication, influencing and negotiating, analysing information and solving problems, and working in and leading teams.

In recent years, the hospital CFO position has emerged in China. The “Hospital financial system” issued by the Ministry of Finance and the Ministry of Health in China proposes that tertiary public hospitals should establish the position of CFO, while other hospitals may determine the necessity based on their specific circumstances [16]. Hospital CFOs in China must be qualified accountants who are members of either the senior accountant or the certified public accountant classification. Due to a shortage of talent in the position of CFO, many hospitals have not appointed CFOs, and even in hospitals where CFO is present, there are often issues with inadequate fulfilment of their responsibilities.

Since the COVID-19 pandemic, China’s hospitals have faced serious challenges in organizational operations, resource allocation and efficiency [17], leading to an increase in the demand of capable CFOs. To address this problem, China’s central government has published several documents, such as “Opinions on accelerating the construction of chief accountant system in tertiary public hospitals” [18] and “The guidelines opinions of building modern hospital management system” [19], to highlight the importance of CFOs and offer further guidance in fostering their managerial competencies. However, there is limited research devoted to understanding the competencies required by hospital CFOs.

In China, as reported by China Association of Chief Financial Officers, the responsibilities of hospital CFOs are primarily reflected in the following: assisting the hospital director in achieving the overall goals of the hospital, supervising and overseeing the responsibilities of the financial department, coordinating with various departments to ensure diligent compliance with financial regulations, and participating in the analysis, recommendations, and decision-making processes related to significant financial matters within the hospital [20]. Hospital CFO faces a range of different expectations from their stakeholders and are expected to fulfil various managerial functions [21, 22]. Specifically, CFOs are generally expected to be involved in organizing and planning financial and operation management, revealing risks, providing suggestions and financial information to support decision-making, and supervising hospitals to implement the relevant national laws and regulations [23–25]. CFOs also play a pivotal role in identifying opportunities and threats in strategy management and take an active part in strategy formulation [26, 27]. Several studies have stressed the urgency of developing managerial competencies to achieve efficient and effective management among China’s hospital CFOs [28, 29]. Additionally, other members of the executive team in China’s hospitals are medical experts and commonly lack management knowledge and skills [30], resulting in an increased demand for professional CFOs in the hospital system. This further reinforces the importance of CFO competency to meet the requirements of fulfilling management functions in daily management.

Competency is typically described as a set of knowledge, skills, abilities and other characteristics necessary for people to meet a range of job needs more effectively than others [31–34]. In terms of human resource management, the competency framework is fundamental to assessing the demands within a specific organization, evaluating performance assessments and monitoring career development [35, 36], as well as providing guidelines for individual self-development [37–39]. Hitherto, there has been limited research on hospital CFOs in the Chinese context. The unexplored areas in the literature indicate a lack of understanding of CFO positions and suggests that CFOs may be poorly prepared for management roles. The competency of CFOs in China’s hospitals have not been widely researched; in this paper, we pursue this challenge.

### Aim

This paper aims to identify specific competencies necessary for China’s hospital CFOs then develop a competency framework of hospital CFOs to fulfil their responsibilities and achieve organization objectives in the Chinese health services context. It will develop our understanding of professional development needs of health service management in China.

## Methods

**Fig. 1:**
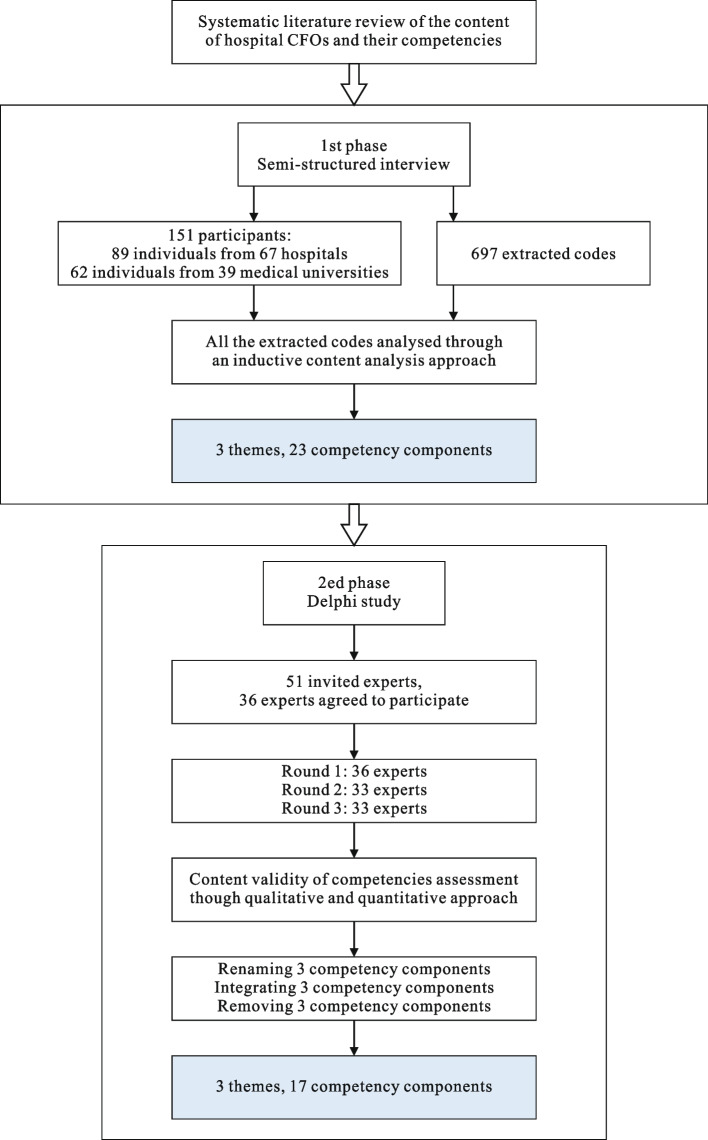
Multi-stage process

Hitherto, the competency of CFO remains an underexplored topic in the literature; hence, a qualitative approach was considered the most suitable method for this research. This research was a cross-sectional, in-depth interview based and descriptive study. Figure 1 displays the process of the multi-stage method.

### Participants

From both theoretical and practical perspectives, we considered public hospital executives and CFOs as well as researchers focusing on hospital management research to be relevant in this study. The hospital executive is the chief executive officer (CEO) who is responsible for nominating candidates for the position of CFO, thus possessing a clear understanding of the competencies required for the role to aid in making appropriate personnel judgment. Researchers focusing on the health system, health economics and hospital management have a deeper exploration of the combination of theory and practice in hospital management. They also possess in-depth and specific knowledge of competencies that enable CFOs effectively manage hospital financial operations. Candidates are selected in accordance with the following four principles: 1) hospital candidates are from tertiary hospitals and have more than 10 years of management experience; 2) researchers come from medical universities and have been engaged in health system or hospital management research for more than 5 years; 3) the geographical distribution of candidates covers different provinces; 4) we are able to establish contact with them; and 5) we tried to split the percentage of respondents from academia and practice equally but adjusted for the actual situation. We invited 210 potential interviewees comprising 124 CEOs and CFOs from 82 hospitals and 86 researchers from 52 medical universities. A total of 71.90% (151 individuals, dram from 15 provinces across mainland China) of them responded to the invitation to participate. The final sample comprised 89 individuals from 67 hospitals, and an additional 62 individuals from 39 medical universities. Of the 67 hospitals included in this study, all were tertiary hospitals (AAA, the highest level of hospital certification), and 88.06% (59 hospitals) were large, with more than 4000 beds. Most respondents (115, 76.16%) were males, and the average age was 45.79 years. Ph.D degree holders were larger in number, with 103 (68.21%). Table 1 reports the demographic characteristics of the study participants.

**Table 1:** Demographic characteristics of participants

Demographic characteristics	Number	Percent
**Gender**		
Male	115	76.16%
Female	36	23.84%
**Educational Status**		
Bachelor	2	1.33%
Master	46	30.46%
Ph.D	103	68.21%
**Profession type**		
Researcher	62	41.06%
Hospital executive	39	25.83%
Hospital CFO	50	33.11%
**Age**		
30–40	55	36.42%
41–50	53	35.10%
51–60	32	21.19%
Above 60	11	7.29%

### Data collection

To explore and develop indicators and a framework of competency for CFOs in China’s hospitals, we adopted in-depth interview (semi-structured interviews) to collect data. At the beginning of interview, researchers introduced the study and participation in the interview, including assurances of anonymity and confidentiality, and answered any questions. Interviews was conducted at the workplace in a conference room during working hours. All interviews were audio recorded, transcribed and checked by interviewers. Interviews were conducted between December 2020 and January 2022, and the average length was approximately 50 min each.

The semi-structured interviews were conducted using a topic guide that outlines the topics to be covered and the main question to be asked. The interview involved questions about “How do you understand the CFO position in China’s hospitals?” and “What knowledge, skills, attitudes, values and traits are necessary for hospital CFOs to practice and fulfil their position responsibilities and requirements?”.

### Data analysis

The original research was intended to focus on application rather than theoretical construction [40]; therefore, a thematic description was considered the most appropriate analysis for the original study [41]. All interview transcripts were analysed using thematic analysis and coded in NVivo. The coding framework was informed by a priori knowledge reflected in the topic guide, initial readings and preliminary coding of the transcripts [40]. Peer debriefing (extensive discussion of transcript data meaning and codes)was undertaken by the team. After the initial coding of five interviews, two researchers coded the same transcripts separately to ensure consistency in the interpretation of the data. Finally, the initial thematic template containing three themes and 23 items was developed, as presented in Table 2.

**Table 2:** Initial thematic template of hospital CFO competencies

Themes	Items
Personality	Honesty and trustworthiness
	Responsibility
	Integrity
	Social responsibility
	Patient services care
	Employee benefits care
Leadership competencies	Planning and organizing
	Critical thinking
	Performance measurement
	Learning
	Creativity
	Communication
	Managerial courage
	Analytical thinking
	Flexibility
Managerial competencies	Expertise
	Strategy management
	Financial budget and program analysis
	Budget formulation
	Knowledge sharing
	Risk management
	Change management
	Technical proficiency

### Delphi process

We applied a Delphi study, which is a systematic research method that uses the judgement of an expert panel, to reach consensus. The criteria we employed to develop a list of potential panel members were academic qualifications, experience in operating hospital or studying hospital management, and level of appointment (e.g., professor, hospital CEO or CFO). We sent email invitations to a selected group of academics and practitioners, providing them with information about the study’s purpose and the estimated time commitment expected from their participation. Out of 51 invited experts, 36 (70.59%) agreed to participate in the Delphi study. The 36 experts originated from 13 different provinces and have an average of 17.27 years of experience in hospital management.

The Delphi study was conducted in three rounds comprising one qualitative and two quantitative rounds. Delphi round 1 was completed by 36 experts, and three experts withdrew due to unexpected unavailability to attend, resulting in a response rate of 91.67% in rounds 2 and 3. Table 3 displays the Delphi study round overview.

**Table 3:** Delphi study round overview

Demographic characteristics	Round 1	Round 2	Round 3
Gender			
Male	23	21	21
Female	13	12	12
Educational Status			
Bachelor	0		
Master	10	9	9
Ph.D	26	24	24
Profession type			
Researcher	20	19	19
Hospital executive	9	8	8
Hospital CFO	7	6	6
Years of working			
10—15 years	10	9	9
15—20 years	13	12	12
Above 20 years	13	12	12

### Delphi rounds

In round 1, the initial template (as reported in Table 2) was sent to the panel, who was required to modify key themes and potential components obtained from the interviews. Two open-ended questions were set as follows: 1) Please kindly provide comments or modifications regarding the proposed themes and components in relation to the competencies expected of hospital CFO; 2) Please add additional competencies that are crucial but missing from the proposed template. The responses were collected and used to generate a comprehensive set of modified and newly proposed components encompassing all themes.

In round 2, self-administered questionnaires were developed based on results derived from round 1. The questionnaire used a five-point Likert scale (1 = strongly disagree; 2 = disagree; 3 = undecided; 4 = agree; s = strongly agree), and experts rated the extent to which they agreed or disagreed with those competencies required by CFO to fulfil position responsibilities.

In round 3, we provided feedback on the results obtained from round 2. The same questionnaire and the feedback as a simple statistical summary of responses from round 2 were sent back to the same group. Experts were encouraged to review and reconsider their initial voting based on the consolidated results. This facilitated the opportunity for members to modify their responses in consideration of the collective opinion with the group.

Consensus was determined by considering the percentage of participants who either “agree” or “strongly agree” with the recommendation statements in both rounds 2 and 3. Following previous studies (e.g., [42]), consensus was defined as a threshold of over 70% agreement among the participants. The data analysis process involved two main steps. First, the comments from round 1 were manually analysed using thematic analysis. Second, standard descriptive statistical analysis for data obtained from rounds 2 and 3 was conducted using SPSS. The reliability and coherence between components in questionnaires were assessed independently using Cronbach’s alpha coefficient. The calculated coefficients ranged from 0.85 to 0.97, indicating sufficient reliability and inter-item correlation.

According to the meaningful comments that emerged from Round 1, several components from the initial template were renamed and integrated. For example, three items were renamed (e.g., ‘personality’ was changed to ‘personal attitudes’; ‘responsibility’ was changed to ‘dependability’; ‘knowledge sharing’ was changed to ‘knowledge management’). Several items were integrated (e.g., ‘patient services care’ and ‘employee benefits care’ were transformed to ‘compassion’; ‘financial budget and program analysis’ and ‘budget formulation’ were transformed to ‘budget and fiscal management’; ‘strategy management’ and ‘change management’ were integrated as ‘applied strategic thinking’). After rounds 2 and 3, three items in total were excluded, namely, managerial courage, performance measurement and technical proficiency. Considering this expert vetting, eventually, the competencies framework of the hospital CFO was established, covering three themes and 17 items.

### Ethical considerations

All participants in the in-deep interviews and the Delphi process gave informed consent in response to a letter that explicitly stated that participation was voluntary and guaranteed complete confidentiality.

## Results

In China, the prerequisite for becoming a hospital CFO is to possess a senior professional and technical qualification in finance or accounting or to have a certified public accountant qualification and work in financial accounting for five years or more. In addition to professional qualifications, hospital CFOs also require a range of competencies to meet the demands of job responsibilities. By interviewed 151 participants from 15 Chinese provinces and adopting a Delphi study with three rounds, we identified three themes in terms of personal attitudes, leadership competencies and managerial competencies, involving 17 items, to develop competency framework for hospital CFOs. Table 4 reports the proposed competency framework necessary for CFOs to fulfil their responsibilities and achieve objectives in China’s hospitals. It also presents statistics for each item in Round 2 (in brackets) and Round 3, and the results of testing the statistical significance for the differences in the mean scores in the two rounds are also reported in Table 4.

**Table 4:** Competency framework for CFO from the Delphi study

Themes	Items	Mean
Personal attitudes	Honesty and trustworthiness	4.44^***^ [4.21]
	Dependability	4.48^***^ [4.17]
	Integrity	4.30^***^ [4.09]
	Social responsibility	4.61^***^ [4.21]
	Compassion	4.34^***^ [3.96]
Leadership competencies	Planning and organizing	4.26^***^ [3.87]
	Critical thinking	4.30^***^ [4.26]
	Learning	4.56^***^ [4.17]
	Creativity	4.22^***^ [3.87]
	Communication	4.74^***^ [4.21]
	Analytical thinking	4.65^***^ [4.13]
	Flexibility	4.39^***^ [4.26]
Managerial competencies	Expertise	4.48^***^ [3.96]
	Applied strategic thinking	4.70^***^ [4.35]
	Budget and Fiscal management	4.65^***^ [4.26]
	Knowledge management	4.22^***^ [3.96]
	Risk management	4.17^***^ [4.04]

It reports the mean values in Round 3 first and those in Round 2 in brackets; a t test was used to test whether the means of competencies of Round 3 have statistical significance compared with Round 2.

^***^ denotes that the mean value is different between rounds with statistical significance levels of 1%

### Personal attitudes

This theme describes individual traits and personality required to determine CFOs’ professionally and ethical behaviour. It comprises five items: honesty and trustworthiness, dependability, integrity, social responsibility and compassion. Participants suggested that this theme is fundamental to the professional attitudes of CFOs to participate in hospital management.

#### Honesty and trustworthiness

CFOs should promote a climate of openness and honesty as well as enabling building trust with organizational members and stakeholders. Participants mentioned that by only building trust with employees, the strategy proposed by CFO can be implemented by organization members. In addition, the supervisory role played by COF also require them to maintain honesty and trustworthiness:

*As a member of the hospital executive team, a CFO has the responsibility to supervise the hospital’s operation management and financial behaviours. A CFO, as a moral modelling for financial personnel, must create an honest atmosphere and ensure the authenticity of financial information. (Participant 3)*

#### Dependability

The CFO is expected to take full responsibility in his or her position, to deal with problems quickly, to display a strong commitment to organizational success and to demonstrate a commitment to delivering on his or her public duty. As participants noted, CFO plays multiple roles in hospital management and requires taking his or her responsibilities seriously and consistently meeting the public’s expectations for health care development and professionalism:

*The functional roles of the CFO refer to developing organizational strategy, allocating resources, monitoring process and evaluating performance. We expect CFOs to hold themselves and others accountable for making principled decisions. (Participant 11)*

#### Integrity

It refers to the CFO behaving in an ethical manner and treating others fairly and with respect. Participants emphasized that hospitals, as providers of health care services, are faced with more ethical choices; therefore, CFOs need to identify ethical dilemmas and conflicts of interest and take action to prevent them when developing organizational strategies. Moreover, CFO should remain fair and objective when communicating with employees:

*The CFO is the value manager of organizational resources, and his or her actions directly affect the development of the organization and the interests of stakeholders; hence, the CFO should remain fair and objective in the allocation of resources and projects. (Participant 7)*

#### Social responsibility

CFOs must be aware of social responsibility undertaken by the hospital and the self, ensure that the decisions and actions made are based on the public interest, and rationally used social resources to improve the efficiency of the hospital. Participants mentioned that CFOs should establish plans and programs for satisfying the needs and expectations of public health care services:

*Hospitals are non-profit organizations responsible for providing health care service to the public, undertaking the social responsibilities of participating in the rescue of major disasters and accidents, and managing public health emergencies. As a member of the executive team of the hospital, the CFO should formulate a development strategy that complies with social responsibility of the hospital. (Participant 9)*

#### Compassion

It describes genuinely caring about others, being helpful when others are in trouble, and showing genuine empathy for others’ emotions. CFOs should understand the emotional components of working in the hospital context and have the remarkable ability to empathize with and demonstrate healthy concern for others. Specifically, compassion emphasizes that CFO pays attention to employee benefits care and patient services care. As participants mentioned:

*Every hospital employee must have a strong sense of empathy, being able to understand the situation of others, and know to put himself or herself in others’ shoes. We expect capable CFOs to put others’ feelings first when necessary and provide help when people approach a problem. (Participant 12)*

### Leadership competencies

This theme refers to the abilities and skills to lead, direct and inspire others to achieve organization goals through teamwork and collaboration, as well as the abilities to motivate and support others to self-development. It comprises seven items: planning and organizing, critical thinking, learning, creativity, communication, analytical thinking and flexibility.

#### Planning and organizing

This references ways CFOs are able to plan and organize daily works, using goal setting, creating work schedules and work plans with associated budgets and resources to achieve established goals. Participants indicated that a capable CFO could integrate resources and apply “best practices” to improve effectiveness and efficiency:

*CFOs should carry out strategic planning and financial planning for the development of the hospital and participate in major decision-making of the hospital; hence, they need abilities to organize work, set priorities and determine resource requirements. (Participant 27)*

#### Critical thinking

It refers to the ability to consider situations from multiple perspectives and to systematically analyse and organize parts. Participants stressed that CFOs can provide creative solutions and identify the link between action and outcomes. A competent CFO can assess the quality of evidence and reasoning, as well as consider the big picture and impact on results:

*In China’s hospitals, most of executive teams are medical experts, and they are professional in the medical field rather than in hospital management. As the professional manager, a CFO must critically compare different points of view and examine situations from multiple or different perspectives when making decisions. (Participant 33)*

#### Learning

It describes the ability to learn actively and continuously, to summarize past achievements and failures to improve productivity, and to identify technological changes and update information reserves. Participants emphasized that the functional role of CFOs involves multiple aspects, such as finance, accounting, strategy and operations, hence, requiring him or her to be able to continuously learn and improve to meet the demands of the position. Without the learning ability, the CFO makes decisions from a single perspective:

*Medicine refers to fields of technological and innovation and require the most cutting-edge knowledge and information. If CFOs do not have the ability to lead and understand the latest medical technology and trends, they will be unscientific when formulating hospital strategies. (Particiapant 37)*

#### Creativity

It refers to the ability to generate original and unique ideas, connecting previously unrelated concepts, to address complex challenges. Participants mentioned that a CFO needs to develop new methods/procedures/processes that are proven to work and encourage new ideas and motivate others to be proactive and resourceful. They highlighted:

*CFOs are different from financial director; they need to work creatively, continuously promote the improvement of hospital operation and processes in accordance with the principle of maximizing benefits, and break the convention to find more effective ways to allocate and use resources. (Participant 2)*

#### Communication

It refers to listening respectfully to others to gain a full understanding of issues and to ensure that important managerial information is shared with employees and others in the appropriate way. Participants emphasized CFOs should communicate clearly and effectively and have responsibility to understand others. A competent CFO can apply skills to present information, analysis and opinions to audiences in a clear, accurate and compelling manner:

*CFOs should not only pay attention to establishing contacts with the financial team, executive team and other departments within the hospital but also be good at establishing contacts with higher authorities, relevant units inside and outside of the health care system and other peers and coordinate the relationship between all parties according to the needs of the work. (Participant 40)*

#### Analytical thinking

This describes the ability to identify and define problems, analyse and verify the cause of the problem by extracting key information, and develop feasible solutions to address the identified problem. Participants indicated that a competent CFO could analyse the situation of the hospital, identify the problem affecting the development of the hospital and select the most effective solution to solve the problem. They noted that:

*The CFO is responsible for making logical deductions from information and identifying a number of solutions to complex problems integrating findings from several different disciplines. (Participant 41)*

#### Flexibility

This refers to the ability to recognize and accept change and new information and rapidly adapt to changing conditions. Participants stressed that CFOs should be aware of the requirements of different situations and be able to effectively adjust their actions to adapt to changing environment. Moreover, CFO is responsible for providing new approaches or solutions to meed the different expectations in changing organizational environment:

*COFs should have the ability to change plan based on input from staff and stakeholders and adjust organizational priorities quickly as situations change. (Participant 31)*

### Managerial competencies

This theme refers to the ability and expertise needed by CFOs to fulfil their responsibilities to ensure that they make more professional judgments and choices. It includes expertise, applied strategic thinking, budget and fiscal management, knowledge management, risk management and problem solving.

#### Expertise

This describes that the needs of a CFO to have professional knowledge in term of finance, accounting, medical system, legal system, rules and regulations to meet the different demands of the position. Participants emphasized expertise contribute to CFO consideration of organizational strategy from a big picture. They highlighted:

*CFOs should be familiar with the professional knowledge in terms of operation management, accounting, finance, law and information technology. Meanwhile, they should understand the development of the health industry and economic society. (Participant 1)*

#### Applied strategic thinking

It refers to the ability to interpret health care policies and analyse organization strategy, as well as to design change management to implement strategic and political directions. Participants stressed that the CFO is responsible for designing for designing performance measures and determining the resource allocation to achieve strategy. They emphasized that:

*CFOs should have a long-term vision to formulate hospital strategic goals and plans while making overall plans and allocating resources to achieve those goals. (Participant 5)*

#### Budget and fiscal management

This describes the ability to plan the work-unit budget, to monitor and evaluate the financial procedures and systems, and to provide suggestions on financial condition. Participants described a capable CFO should develop, monitor and evaluate procedures and standards to ensure the good financial condition of the hospital. Participants also emphasized that a CFO needs to analyze budget statements and financial reports while making recommendation to improve the financial performance of hospitals:

*One of responsibilities for the CFO is to use financial and other quantitative information to manage the hospital and understand overall financial performance of hospital, as well as analyze financial information to evaluate strategic opportunities and options. (Participant 51)*

#### Knowledge management

It refers to the ability to identify and provide useful information to others to increase organizational effectiveness. Participants highlighted the CFOs can collect and obtain external and internal information through novel approaches and then share that information with others in a timely manner to reduce information asymmetry. They stated that:

*CFOs are expected to plan, develop and manage information storage and retrieval systems and to promote organizational development by sharing knowledge and information. (Participant 43)*

#### Risk management

It refers to the ability to plan and implement measures to avoid, overcome or compensate for risk factors. Participants emphasized that CFOs should manage work and information within a strategic framework, establish a proven approach to risk management, identify risks of negative outcome, and develop solutions to reduce risk and maximize value. A capable CFO can communicate the impact of identified risks and apply corrective action. Participants stressed the following:

*CFOs should carefully sort out and manage the operation process of hospitals to ensure effective management of various risks for hospitals. (Participant 20)*

## Discussion

Emerging evidence highlights the significant role played by CFOs in improving hospital operation and management [14], especially during the COVID-19 pandemic. A growing number of public hospitals in China have established the position of CFO; however, competent hospital CFOs are scarce. Like CFOs in the NHS who must be qualified accountants who are members of either the Chartered Institute of Management Accountants (CIMA) or a member of one of the five accountancy institute members of the Consultative Committee of Accountancy Bodies, hospital CFOs in China need to hold the professional qualification of senior accountant or certified public accountant. The responsibilities of hospital CFOs are complex and varied, such as protector, supporter, innovator and strategist [24], thus requiring various competencies to fulfil position responsibilities.

This study aims to explore competencies necessary for management practice by CFOs in China’s hospitals and develop a competency framework for hospital CFOs. Using a qualitative research method, by interviewing 151 participants (hospital executive, CFO and researcher) from 15 Chinese provinces, we identified the initial thematic template covering three themes and 23 competencies. Subsequently, we applied a three-rounds Delphi process to verify expert validity. The ultimate competency framework for hospital CFOs composed of 17 competencies and organized into three themes: personal attitudes, leadership competencies and managerial competencies. The proposed framework highlighted that fulfilling complex responsibilities in hospital management practice requires the integration of different competencies.

This competencies framework highlighted the role played by hospital CFOs and their responsibilities proposed in previous studies. For example, competencies such as social responsibility and dependability reflect that the hospital CFO should fulfil a position based on the organization’s mission and expectations, which is consistent with Bish and Becker [43], who stated that leadership and management are different in nonprofit, as nonprofits have unique strategic and operational characteristics. Critical thinking, analytical thinking, creativity and flexibility may be indicators of the growing emphasis of CFOs involved in formulating and executing organizational strategy [44]. Furthermore, the competencies of communication and flexibility encourage hospital CFOs to lead others to achieve objective in a more effective way [45]. The ability of budget and fiscal management indicates the growing emphasis on the role played by CFOs in the accountability and financial performance of hospitals. The element of risk management reflects CFO’s responsibilities, such as advising on project evaluations both for financial and nonfinancial impacts, forecasting based on financial performance, identifying risk and providing suggestions [46].

In the comparison with the competency models in other countries, there are some similarities. The previous competency framework for public CFOs [47] emphasized the domains of strategic and financial, which were similar to our results. Additionally, the description of the NHS CFO position highlights the value in terms of compassion and integrity, essential CFO attributes such as planning, communication, flexibility and analytical thinking [15]. Our study also emphasized the competencies necessary for China’s hospital CFOs to fulfil their responsibilities. Additionally, our proposed framework expanded the theme of personal attitudes of CFOs by adding dependability, honesty and trustworthiness, which are commonly valued in Chinese culture.

This paper provides several theoretical contributions and practical implications. First, this qualitative research contributes to deeply exploring the competency required by CFOs in China’s hospitals. Gaining the insights and perspectives of CFOs currently working in hospital sector expands our understanding and knowledge of competency requirements in an understudied context. The findings indicate that the uniqueness of hospital affect the requirements of CFO competency, which will have an impact on organizational processes in terms of recruitment, selection and human resource management. In practical terms, the proposed competency framework clarifies the management requirements of hospital CFOs and can be used as the cornerstone for establishing selection criteria, guidance tools and development programs. Finally, the analysis of the required competencies for CFO provides significant practical implications for designing human resource management practices for hospital management in China.

This study has some limitations and provides opportunities for future research to strengthen and develop our findings. As the qualitative approach was adopted in this study, this research was limited by the sample size (*N* = 151, from 15 Chinese provinces), which may limit the comprehensive identification of competencies required for CFO practice in China’s hospital. Furthermore, the extent to which we can generalize these findings to all hospitals in China is limited, and our findings need to be validated in other hospital settings (e.g., secondary hospitals and private hospitals). It seems the good opportunities for future research. Compared with our cross-sectional design, using a longitudinal design would provide stronger evidence of the expected competencies requirements of CFOs over time. The proposed competency framework was conducted based on respondents’ perceptions of the expectations of their CFOs. Future research could consider adding the objective measured of performance expectations for CFO position to enhance this research. Additionally, it cannot be overlooked to develop assessment approaches that can be used to assess the competency of the current hospital CFO in China, and future research could fill this gap.

## Conclusion

The role played by hospital CFO in strategy, performance and risk management is gaining increasing attention from practitioners and scholars. China’s hospital seeks competent CFOs to take on their responsibilities, especially in an increasingly complex organizational environment. This paper provides insights into the competencies required by CFOs to achieve their responsibilities. The proposed framework identifies the integration of different competencies in terms of personal attitudes, leadership competencies and managerial competencies as the core competencies for CFO in hospital management. It expands an underexplored topic in prior studies and provides a systematic framework to develo hospital CFO in China, including establishing clear management competency requirements to guide management position recruitment, development and performance management.

## Data Availability

The data and materials analysed during the current study are not publicly available because they are part of datasets for three further papers, but the interview materials (questions) are available from the corresponding author on reasonable request.
